# Designing high-performance polypropylene *via* synergistic free radical scavenging-intumescent flame retardancy: excellent mechanical performance and flame retardancy

**DOI:** 10.1039/d5ra05200a

**Published:** 2025-09-22

**Authors:** Xiao-han Zhou, Xiao-ling Zang, Tian-chao Shi, Jing Xie, Ping-li Wang, Jun-hui Ji, Ge-xia Wang, Dan Huang, Xu-ran Liu, Zhi-chao Zhen

**Affiliations:** a College of Materials Engineering, North China Institute of Aerospace Engineering Langfang Hebei 065000 China liuxuran15@mails.ucas.ac.cn; b National Engineering Research Center of Engineering and Ecological Plastics, Technical Institute of Physics and Chemistry, Chinese Academy of Sciences Beijing 100190 China danhuang@mail.ipc.ac.cn zhenzc@mail.ipc.ac.cn; c College of Chemistry and Materials Engineering, Beijing Technology and Business University Beijing 100048 China; d Municipal Commission of Science and Technology Beijing 101117 China

## Abstract

Polypropylene (PP) faces application limitations due to its inherent flammability. Although halogen-free intumescent flame retardants (IFRs) are environmentally friendly, they often suffer from low flame-retardant efficiency, poor compatibility, and mechanical performance deterioration. Herein, based on a two-phase synergistic flame-retardant mechanism, a highly reactive flame-retardant synergist-1,3-bis(2-acrylamidoethylamino) propane (BAAEP) was designed, and combined with the IFR system including ammonium polyphosphate (APP) and triazine-based char-forming agent (CFA) to achieve synergistic flame retardancy in PP. The multiple active groups in BAAEP (amide groups, C=C double bonds, and amino groups) catalyzed crosslinking and char formation in the PP matrix while simultaneously scavenging gaseous free radicals. Furthermore, combined with the intumescent charring effect of IFR, a physical and chemical synergistic flame-retardancy mechanism was established, achieving the integration of high-efficiency flame retardancy and smoke suppression. At the same time, compared to pure PP, there was no significant decrease in thermal or mechanical performance. The optimal flame-retardancy effect was achieved when the addition amount of BAAEP was 4‰. The peak heat release rate (PHRR) of the composite material decreased by 74.3%, total smoke release (TSR) decreased by 83.9%, the vertical burning test (UL-94) rating reached V-0, and the limiting oxygen index (LOI) increased to 32.8%. This work presents a novel strategy for developing high-performance PP with integrated flame retardancy, smoke suppression, and mechanical performance.

## Abstract 2

A reactive synergist (BAAEP) was designed for PP, paired with IFR(APP/CFA) for two-phase flame retardancy. It catalyzed charring, scavenged radicals, and synergized with IFR's intumescent effect for efficient flame retardancy and smoke suppression.

## Graphical abstract

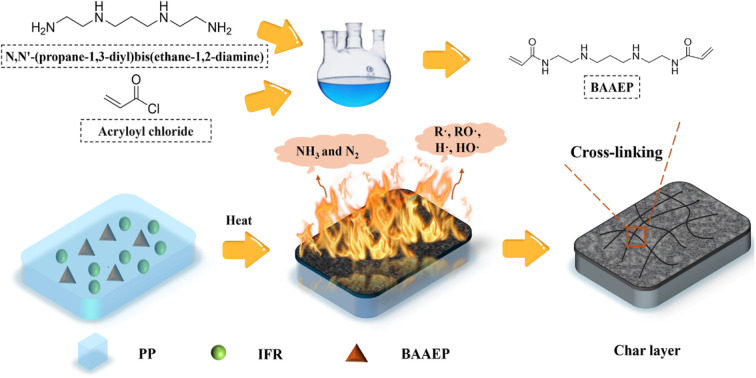

## Introduction

Although PP is one of the most widely used general-purpose plastics, possessing excellent processing performance and cost advantages, it has serious fire hazards due to its flammability (with a LOI of only 17–18%), which restricts its further application.^1^ Adding flame retardants to the polymer matrix is the most direct and effective method to reduce the flammability of PP. Currently, mainstream halogen-free IFRs utilize the synergistic effects of carbon sources (such as CFA, pentaerythritol, *etc.*),^2^ acid sources (such as APP, ammonium pentaborate, *etc.*),^3,4^ and gas sources (such as melamine, dicyandiamide, *etc.*) to form an intumescent char layer during combustion, which provides both barrier and smoke suppression functions.^5,6^ However, to achieve excellent flame retardancy, a loading of 25–30 parts is usually required to meet the UL94 V-0 rating, which not only significantly degrades the mechanical performance of the material but also poses challenges in processing and increases costs.^6–8^ Therefore, the development of novel flame retardants with high efficiency and low loading has become a key challenge in the flame retardant modification of PP.

Adding flame retardant synergists is a commonly used method to improve the efficiency of IFR flame retardants and enhance mechanical performance. Currently, the commonly used flame retardant synergists for PP mainly include inorganic minerals such as wollastonite,^9^ montmorillonite,^10^ and halloysite,^11^ metal oxides, 4A molecular sieves,^12^ and silicon-containing compounds.^13,14^ They primarily reduce the amount of IFR added by promoting char formation, but they struggle to significantly improve the decline in thermal and mechanical performance of the material. Kong *et al.* improved the flame retardancy of composites by adding the modifying Fe-montmorillonite (Fe-OMT) as flame retardant synergists, while also reducing the amount of MH added to the composites. The results showed that when the addition amount of Fe-OMT exceeded 5%, the flame retardancy rating reached V-1, but it also began to cause deterioration in thermal performance. This indicates that in order to achieve both mechanical and flame retardancy performance, it is crucial to develop a new synergist that enhances synergistic flame retardancy.

The flammability of PP stems from the fact that the tertiary hydrogen in its main chain is easily broken at high temperatures to produce alkyl/hydroxyl radicals, which trigger a chain combustion reaction.^15^ Therefore, blocking the chain radical reaction of PP is the key to controlling PP combustion. Traditional synergistic flame retardants can promote char formation but lack sufficient efficiency of flame retardancy, while flame retardants based on the radical scavenging mechanism (such as halogen-based) are efficient but pose environmental concerns. Therefore, designing a novel halogen-free synergist that can efficiently capture and quench the active free radicals generated during the combustion of PP can fundamentally achieve PP flame retardancy from the source, which is rarely reported in current research.^16,17^

Based on the different mechanisms of action of radical scavengers during combustion, they can be divided into gas-phase radical scavengers and condensed-phase radical scavengers.^18^ In the early stages of thermal oxidative degradation, polymer materials initially degrade into chain radicals in the condensed phase, while there are no highly reactive radicals in the gas phase. Some carbon nanomaterials (such as fullerenes,^19,20^ graphene,^21,22^ carbon nanotubes^23,24^), inorganic phosphorus-based flame retardants (such as ammonium polyphosphate),^25^ non-volatile organic phosphorus flame retardants (such as phosphonates),^26^ and metal compounds^27^ can capture chain radicals in the condensed phase, inhibiting the initial thermal oxidative degradation of polymers, thereby delaying or inhibiting the progression of combustion. The flame retardants based on gas-phase flame retardant mechanism have relatively high flame retardance efficiency and the flame retardance purpose can be achieved at a lower additive amount, having little impact on the physical and mechanical performance of the material. The most representative of these are bromine and bromine-antimony composite flame retardants, and some volatile organic phosphine flame retardants,^28^ nitrogen compound flame retardants,^29–33^*etc.*, which can capture high-reactive free radicals in the gas phase to generate low-reactive free radicals, stable compounds, inert gas, *etc.*, achieving gas-phase flame retardance. The essence of capturing active free radicals is that it has highly reactive functional groups, such as double bonds, amide bonds, amino groups, *etc*, which is of great significance to design efficient flame retardant synergists.^34^

Based on the dual-phase synergistic flame retardance mechanism, a highly reactive flame-retardant synergist-BAAEP was innovatively designed and synthesized in this work. At the same time, APP was selected as the acid source, and CFA was selected as the carbon source and gas source to form an intumescent flame retardant. The composite flame-retardant PP material was prepared through melting extrusion. To achieve the purpose of BAAEP serving as a free radical scavenger in both the condensed and gas phases, the molecular structure design features the following characteristics: (1) multiple amide groups were introduced into the molecular backbone, and cross-linked networks were formed through reactions between terminal amide groups and polymer free radicals, effectively delaying the thermal decomposition of the material; (2) active functional groups such as C=C double bonds and amino groups were integrated to efficiently capture free radicals in the gas phase, thereby interrupting the combustion chain reaction. The thermal stability and mechanical performance of the flame-retardant material was evaluated through thermogravimetric (TG) as well as tensile and impact tests. The flame-retardant performance of the flame-retardant material was comprehensively assessed using UL-94, LOI, and cone calorimetry (Cone). The synergistic flame-retardant mechanism of BAAEP and IFR was further explored and revealed through tests such as scanning electron microscope-energy dispersive X-ray spectroscopy (SEM-EDX) and pyrolysis gas chromatography-mass spectrometry (Py-GC/MS).

## Experimental

### Materials

PP (99%) was purchased from China Petroleum & Chemical Corporation, Beijing Yanjiao Branch; APP (molecular formula (NH_4_PO_3_)_*n*_, P content of ≥31.0 wt%, *N* content of ≥14.0 wt%, degree of polymerization (DP > 1000), industrial grade) was purchased from Shifang City Changfeng Chemical Co., Ltd; CFA (>99.5%) was purchased from Guangzhou Yinyuan New Materials Co., Ltd; *N*, *N*′-(propane-1,3-diyl)bis(ethane-1,2-diamine) (C_7_H_20_N_4_, AR), acryloyl chloride (AC, C_3_H_3_ClO, AR), acetonitrile (C_2_H_3_N, AR), and methanol (CH_3_OH, AR) were purchased from Shanghai McLean Biochemical Technology Co., Ltd.

### The synthesis of BAAEP flame retardant synergist

A certain amount of acetonitrile was added to a four-necked flask with a stirring paddle and nitrogen gas, and then *N*, *N*′-bis (2-aminoethyl)-1,3-propanediamine and acryloyl chloride with a molar ratio of 1 : 2 were added to the flask. After reacting at low temperature (0–5 °C) for a period of time, the reaction was continued at 30 °C until completion. The resulting product was filtered, washed with acetonitrile, and then dried in a vacuum oven for 12 hours. The product, obtained as a solid powder, was further recrystallized in a methanol/water mixture at −28 °C to obtain the refined product BAAEP.

### Preparation of PP flame retardant composites

PP, IFR (APP : CFA = 4 : 1), and synergist BAAEP were mixed evenly in the ratio shown in Table 1, then added into a twin-screw extruder (Labtech Engineering Co., Ltd, LSF-26) for extrusion granulation. The main engine and feeder speeds were set to 170 r min^−1^ and 25 r min^−1^, respectively, and the temperature ranges of the twin-screw extruder were set to 130, 150, 170, 185, 185, and 170 °C, respectively.

**Table 1:** Formula of PP and PP flame-retardant composites^a^

Sample	Component content (wt%)			BAAEP [g]
	PP	IFR		
		APP	CFA	
PP	100	—	—	—
P1	78	17.6	4.4	—
P2	74	20.8	5.2	—
P3	78	17.6	4.4	1
P4	78	17.6	4.4	2
P5	78	17.6	4.4	3
P6	78	17.6	4.4	4
P7	78	17.6	4.4	5

a The mass of BAAEP is added to 1 kg of PP/IFR.

Standard specimens: the sample particles are prepared by the injection molding machine (Ningbo Shuangsheng Plastic Machinery Co., Ltd, SSF320-G), setting the injection temperature at 190 °C and the injection pressure at 60 MPa.

Sheet: the sample particles were dried in a vacuum oven at 50 °C for 24 h. The dried particles were pressed in a hot press (Labtech Engineering Co., Ltd, LP20-B), preheated at 200 °C for 5 min, and then held at 3 MPa for 2 min, and then cooled to obtain a sheet of uniform thickness.

The samples obtained above were used for relevant characterization and testing.

### Performance testing and characterizations

#### Structural characterization test

Nuclear magnetic resonance (^1^H NMR, Bruker EMX-300) spectrum test: deuterated water was used as the solvent and tetramethylsilane as internal standard for chemical shifts.

Fourier infrared spectrum test (FTIR, INVENIO S): an infrared spectrum with a wavelength range of 4000–500 cm^−1^ was obtained at a resolution of 4.0 cm^−1^.

Element content test (EA, Elementar Unicube): each sample is tested twice in parallel.

#### Thermal stability and mechanical performance test

TG (Hitachi STA200): the temperature was increased from room temperature to 800 °C at 20 °C min^−1^ under nitrogen atmosphere.

Scanning electron microscope (SEM, Hitachi S-4800): the standard tensile specimens were quenched to obtain cross-sections, and the morphology of the cross-sections of PP and its flame-retardant composites were observed.

Tensile performance test (universal material testing machine, Shanghai Songdun WDW-10): according to the GB/T 1040.1–2006 standard, dumbbell-shaped standard tensile specimens were stretched at a rate of 50 mm min^−1^ at 25 °C. The dimensions of the standard tensile specimens were: effective length *G*_0_ = 25 ± 1 mm, width *b* = 4.0 ± 0.4 mm, and thickness *d* = 2.0 ± 0.2 mm. The test results were reported as the arithmetic mean of five samples.

#### Flame retardancy test

The LOI test (TTeech-GBT2406-2): according to the GB/T 2406.2–2009 standard, the dimensions of specimens were150 mm × 6 mm × 3 mm, and the test results were calculated as the arithmetic mean of five samples. V(O_2_) represents the oxygen flow rate and V(N_2_) represents the nitrogen flow rate. The oxygen index LOI was calculated using eqn (1):1
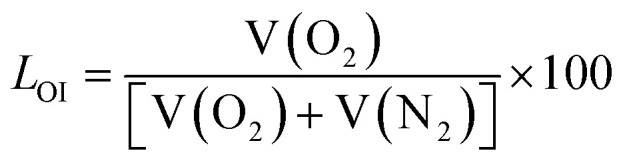


The UL-94 (TTeech-GBT2408): according to the ASTM D2863 standard, the dimensions of specimens were 150 mm × 12.7 mm × 3 mm. The fixed ignition time was set to 2 s, and the test results were taken as the arithmetic average of 5 samples.

Cone test (VOUCH 6810): according to the ISO 5660 standard, the dimensions of specimens were 100 mm × 100 mm × 3 mm, and the heat flux was 35 kW m^−2^.

#### Flame retardant mechanism analysis

SEM (ZEISS GeminiSEM 300)-EDX: the morphology of the flame retardant and he residual char after cone calorimeter testing was observed and analyzed by using SEM; the elemental composition of the residual char was analyzed by using EDX.

Py-GC/MS (pyrolyzer: EGA3030D, GC-MS: GCMS-QP2020NX): pyrolysis was conducted at 600 °C, and the pyrolysis products were searched against the NIST08 s mass spectral library.

## Results and discussion

### Structural characterization of flame retardant synergists

**Fig. 1:**
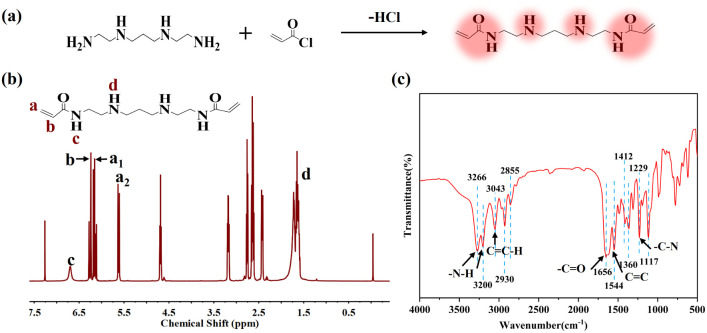
Design route (a) of the flame retardant synergist BAAEP with multiple active functional groups and its ^1^H NMR (b) and FTIR spectra (c).

To achieve the purpose of using the flame retardant synergist as a free radical scavenger in both the condensed and gas phases, a nitrogen-containing compound named BAAEP, which contains terminal amide groups as well as multiple reactive groups such as C=C double bond and amino group, was designed and synthesized (Fig. 1(a)).^34^ The structure of this synergist was verified by nuclear magnetic and infrared analysis, as shown in Fig. 1(b) and (c). In the ^1^H NMR spectrum, the wide peak at the chemical shift of 6.70 ppm is attributed to the more active proton of –NH– in amide functional group, and the characteristic absorption peaks at 6.24 ppm, 6.16 ppm and 5.63 ppm correspond to the protons of CH_2_=CH– in turn. The –NH– proton, which is connected to –CH_2_ at both ends, may cause a characteristic absorption peak at about 1.65 ppm, which may also be due to the incomplete reaction of –NH_2_. In the infrared spectrum, the absorption peaks at 3266 cm^−1^ and 3200 cm^−1^ represent the stretching vibrations of –N–H; the absorption peak at 3043 cm^−1^ is attributed to the stretching vibration of C=C–H; the absorption peaks at 2930 cm^−1^ and 2855 cm^−1^ signify the symmetric vibration of –CH_2_; the absorption peak at 1656 cm^−1^ corresponds to the stretching vibration of –C=O; the peak at 1544 cm^−1^ indicates the stretching vibration of C=C; and the absorption peak at 1229 cm^−1^ represents the stretching vibration of –C–N. The above results show that the flame retardant synergist BAAEP has a variety of the expected active groups. Further elemental analysis of the BAAEP synergist was conducted, and the results are presented in Table S1. The measured oxygen content confirms the formation of the amide bond. However, slight deviations were observed between the measured values of C, H, N, and O and the calculated values calculated for the pure compound (C_13_H_24_N_4_O_2_), indicating the presence of minor impurities.

### Flame retardant PP composite materials

#### Structural characterization

**Fig. 2:**
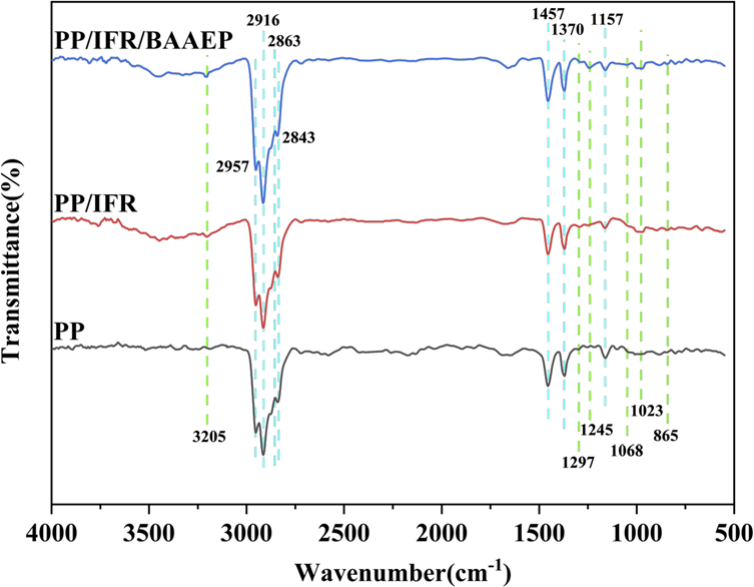
FTIR spectra of PP, PP/IFR (P1) and PP/IFR/BAAEP (P6) composite materials.

As shown in Fig. 2, several of the most common PP characteristic peaks are detected in the infrared spectra of PP before and after flame retardant modification. Among them, the absorbance peaks of 2800–3000 cm^−1^ are assigned to the stretching vibration of C–H (–CH_2_, –CH_3_). The absorption peaks at 1457 cm^−1^ and 1370 cm^−1^ are the bending vibration absorption peaks of –CH_2_ and –CH_3_ of PP, respectively. And the rocking vibration absorption peak of –CH_3_ also appeared at 1157 cm^−1^. After the introduction of IFR, the peak at 3205 cm^−1^ corresponds to the stretching vibration peak of –N–H in IFR flame retardant. The absorption peak of P=O appears near 1068 cm^−1^, and the peaks at 1023 cm^−1^ and 865 cm^−1^ are the characteristic absorption peaks of P–O–C. The appearance of these characteristic peaks clearly indicates that the IFR flame retardant has been successfully added to PP. It is worth noting that after the introduction of BAAEP, in addition to the above characteristic peaks, there is a characteristic absorption peak of –C–N at 1245 cm^−1^, and the characteristic absorption peak of 3205 cm^−1^ –N–H is also more obvious, which proves the successful introduction of BAAEP. Unfortunately, there is no obvious evidence to determine whether the flame retardant interacts with the PP matrix.

#### Thermal stability and mechanical performance

**Fig. 3:**
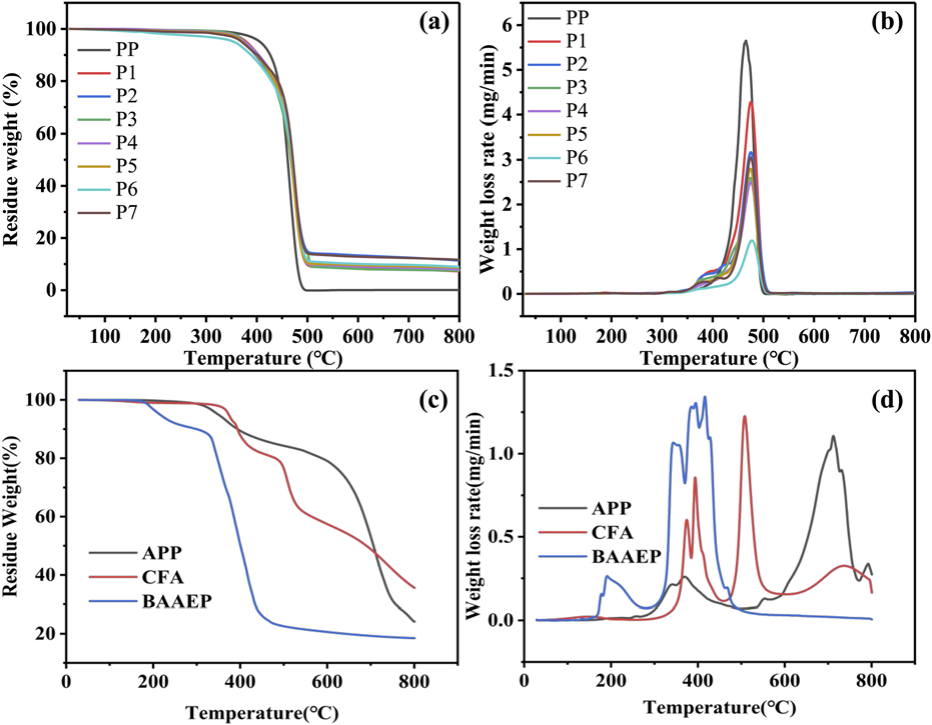
TG (a) and derivative thermogravimetric (DTG) (b) curves of PP and its flame-retardant composites; TG (c) and DTG (d) curves of APP, CFA, and the synergist BAAEP.

The thermal stability test results for PP and its flame-retardant composite materials are shown in Fig. 3(a) and (b) and Table 2. In the pure PP, the *T*_5%_ of is 406 °C, the *T*_max_ is 465 °C, and the char yield (CY) is 0.06%, indicating that PP undergoes nearly complete thermal decomposition. After adding IFR, the CY of the PP composite material significantly increases, with CY values of 7.24% and 11.31% for P1 and P2, respectively, indicating the flame-retardant effect of IFR in promoting char formation. The *T*_5%_ of P1 is reduced to 379 °C, and with the increase in the amount added, the *T*_5%_ is even reduced to 374 °C. This is because IFR decomposes to release non-combustible gases before PP decomposes. IFR undergoes a weight loss stage due to decomposition before 400 °C (Fig. 3(c) and (d)), resulting in *T*_max1_ of PP/IFR being only 398 °C and 384 °C, which are lower than *T*_max_ of pure PP (465 °C). However, during the subsequent heating process, the thermal degradation rate is low, indicating good thermal stability, with *T*_max2_ being 474 °C, which is higher than the decomposition temperature of pure PP. Therefore, the compounding of IFR with PP does not weaken the thermal stability of the flame-retardant composite material.

**Table 2:** Thermal stability parameters of PP and its flame-retardant composites

Sample	*T*_5%_ [°C]	*T*_max1_ [°C]	*T*_max2_ [°C]	Char yield at 800 °C [%]
APP	350	369	712	24.11
CFA	374	394	508	35.52
BAAEP	216	193	416	18.48
PP	406	—	465	0.06
P1	379	398	475	7.24
P2	374	384	475	11.31
P3	376	376	474	7.47
P4	377	373	475	7.97
P5	372	376	474	8.67
P6	355	370	477	8.92
P7	370	382	475	11.64

After adding BAAEP to PP/IFR, the *T*_5%_ and *T*_max1_ of PP/IFR/BAAEP further decrease significantly. When the amount of BAAEP added reaches 4 g, *T*_5%_ and *T*_max1_ drops to their lowest values, with a decrease of 24 °C and 18 °C respectively compared to PP/IFR. This is because, on the one hand, the *T*_5%_ (216 °C) of BAAEP is relatively low, leading to a decrease in the initial *T*_5%_ of PP/IFR/BAAEP; on the other hand, BAAEP also undergoes a weight loss stage due to decomposition at around 200 °C (Fig. 3(c) and (d)), which further results in an overall lower *T*_max1_ for PP/IFR/BAAEP. It is worth noting that *T*_max2_ can maintain a high value, and even the *T*_max2_ of PP/IFR/BAAEP (BAAEP 4 g) is 2 °C higher than that of PP/IFR, indicating good thermal stability. The addition of BAAEP results in a higher CY value for PP/IFR/BAAEP compared to PP/IFR, indicating that BAAEP plays a role in radical scavenging during the thermal decomposition of PP/IFR/BAAEP, thereby enhancing its thermal stability at high temperatures.

In terms of mechanical performance (Table 3), compared with PP, the tensile strength, elastic modulus, elongation at break, and impact strength of PP/IFR decreases in different degrees. The degree of reduction intensifies with the increase in IFR content, which is mainly attributed to the poor compatibility between IFR and the matrix. After adding BAAEP, although the elongation at break of PP/IFR/BAAEP slightly decreases compared to PP/IFR (P1), the tensile strength, elastic modulus, and impact strength show a slight improvement trend. At the same time, it is observed through SEM (Fig. S1) that the brittle cross-section of the PP specimen is relatively smooth, while the brittle cross-section of the PP flame-retardant composite material is coarse and uneven. As the IFR content increases, the unevenness of the brittle cross-section becomes more severe. This is because a higher content of flame retardant is more difficult to disperse in the PP matrix and is prone to agglomeration. Compared with PP/IFR with the same IFR content, the phase separation phenomenon of PP/IFR/BAAEP specimen is relatively improved, which indicates that BAAEP plays a cross-linking role, enhancing the compatibility between IFR and the matrix, and thereby improving the mechanical properties of the material.

**Table 3:** Mechanical properties parameters of PP and its flame-retardant composites

Sample	Tensile strength [MPa]	Elastic modulus [MPa]	Elongation at break [%]	Impact strength [kJ m^−2^]
PP	24.1 ± 2.4	689 ± 42	51 ± 3	3.3 ± 0.6
P1	22.0 ± 2.4	643 ± 201	48 ± 3	3.2 ± 0.2
P2	21.8 ± 1.6	527 ± 131	43 ± 9	2.9 ± 0.4
P3	21.1 ± 1.4	723 ± 178	35 ± 7	3.4 ± 0.1
P4	22.5 ± 1.2	544 ± 40	48 ± 10	3.6 ± 0.3
P5	22.7 ± 1.5	558 ± 56	32 ± 7	3.8 ± 0.1
P6	22.7 ± 1.1	702 ± 237	33 ± 10	3.9 ± 0.2
P7	21.9 ± 1.3	651 ± 175	29 ± 6	3.8 ± 0.5

In summary, compared to pure PP, the introduction of the flame retardant IFR will inevitably lead to a decrease in the material's thermal stability and mechanical properties, and this deterioration effect becomes more pronounced with an increase in the flame retardant content. It is worth noting that when the flame-retardant synergist BAAEP was further added to enhance flame retardancy, this component unexpectedly improved the interfacial compatibility between the IFR and the matrix. This synergistic effect not only compensated for the loss of mechanical properties caused by the addition of the flame retardant but also maintained the thermal stability level of the original PP/IFR composite material.

**Fig. 4:**
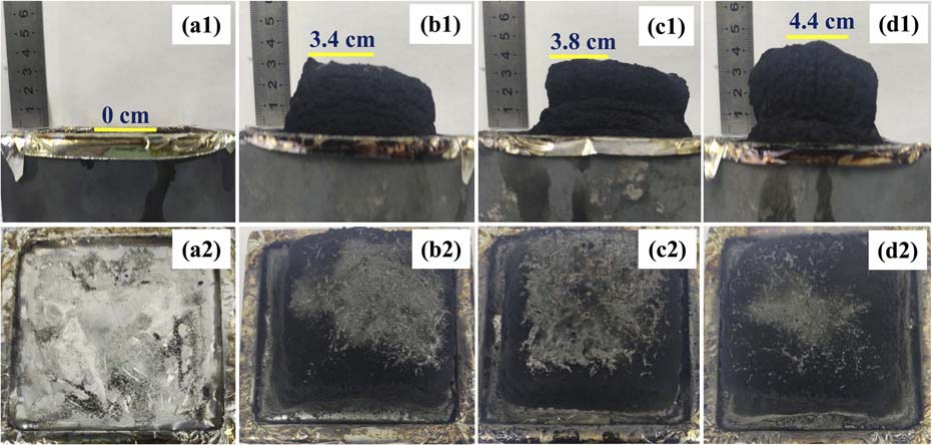
Photographs of char residue after cone calorimeter tests: (a) PP (b) P1 (c) P2; (d) P6.

Flame retardant performance. The LOI and UL-94 vertical burning test are commonly used test methods for evaluating the combustion performance of polymer materials. As shown in Table 4, pure PP exhibits flammable performance with an LOI of only 17.5% and is unrated (NR) in the UL-94 vertical burning test. After adding the intumescent flame retardant, an expanded carbon layer appeared on the surface of the PP/IFR (P1) (Fig. 4). The LOI significantly increases to 23.5%, and the UL-94 combustion rating is improved to V-2, but there is still a dripping phenomenon. Only when the content of the intumescent flame retardant continues to increase, that is, when the ratio of PP: APP: CFA is 78 : 20.8 : 5.2 (P2), *t*_1_ and *t*_2_ significantly decrease, and the dripping phenomenon disappears. And further, the LOI notably increases to 35.7%, with the burning class rising to V-0.

**Table 4:** UL-94 and LOI test data of PP and its flame-retardant composites^a^

Sample	UL-94					LOI [%]
	*t*_1_ [s]	*t*_2_ [s]	Dripping	Cotton ignited	Rating	
PP	—	—	Yes	Yes	NR	17.5
P1	7.6 ± 0.5	31.3 ± 1.3	Yes	Yes	V-2	23.5
P2	1.0 ± 0.1	1.3 ± 0.1	No	No	V-0	35.7
P3	4.1 ± 0.3	2.8 ± 0.3	Yes	Yes	V-2	22.6
P4	3.1 ± 0.3	2.2 ± 0.3	Yes	Yes	V-2	24.6
P5	2.8 ± 0.2	1.9 ± 0.2	Yes	Yes	V-2	25.3
P6	0.9 ± 0.1	1.2 ± 0.1	No	No	V-0	32.8
P7	0.9 ± 0.1	1.3 ± 0.3	No	No	V-0	33.1

a *t*_1_ and *t*_2_ in UL-94 refer to the time from the start of combustion to the complete extinction of the sample after the first and second ignitions in the test, respectively.

To investigate the impact of the introduction of homemade flame-retardant synergist BAAEP on the flame retardancy of PP, we introduced different contents of the flame retardant synergist BAAEP into the PP composite material (PP: APP: CFA = 78 : 17.6 : 4.4) matrix. It can be observed that as the content of the flame retardant synergist BAAEP increases, *t*_1_ and *t*_2_ significantly decrease, preventing the material from continuing to burn and instead allowing the flames to be carried away by molten droplets, thereby improving the material's combustion performance. Specifically, when the BAAEP addition reaches 4 g, the flame-retardancy effect is optimal. After being ignited for 10 seconds, it self-extinguishes upon removal from the flame. Not only does the melting and dripping phenomenon disappear, but the UL-94 burning rating is also upgraded to V-0, and the LOI is increased to 32.8%. It is worth noting that when the amount of BAAEP added is greater than 4 g, the flame-retardancy effect will not be significantly improved.

**Fig. 5:**
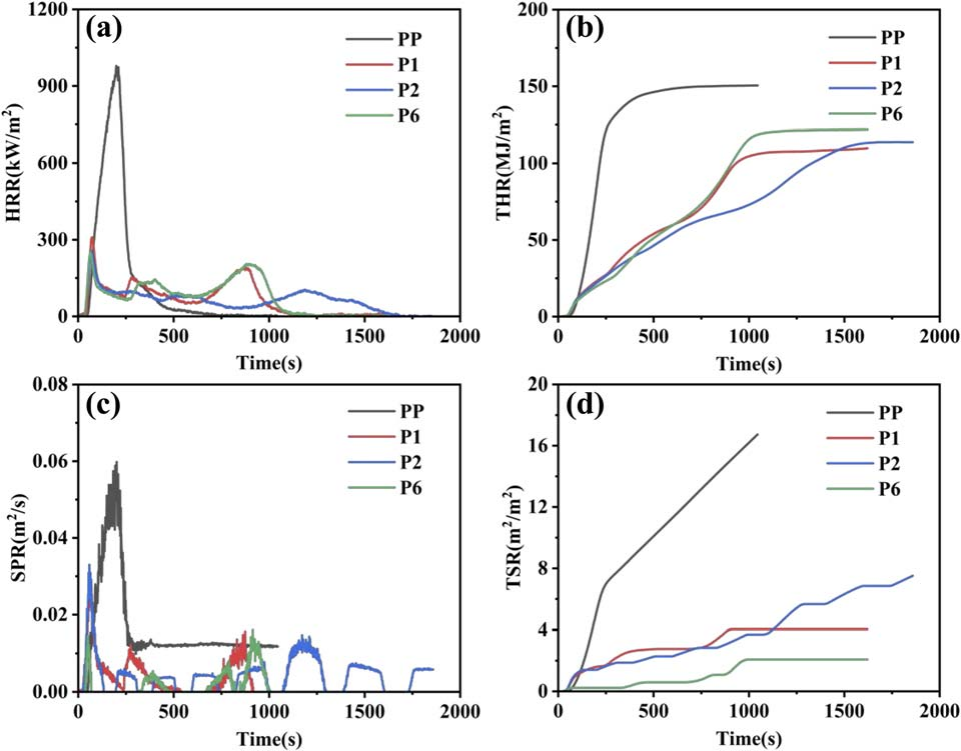
(a) Heat Release Rate (HRR), (b) Total Heat Release (THR), (c) Smoke Production Rate (SPR), and (d) Total Smoke Release (TSR) curves of PP and flame-retardant PP composites.

The cone calorimeter test data for PP and its flame-retardant composites are shown in Fig. 5 and Table 5. It can be seen that the TTI of pure PP is 43 s, PHRR is 978.5 kW m^−2^, *t*_PHRR_ is 200 s, THR is 150.5 MJ m^−2^, FPI is 0.044 s m^2^ kW^−1^, FGI is 4.89 kW (m^2^ s), and TSR is 1451.4 m^2^ m^−2^. After adding IFR, the cone calorimeter test parameters of the P1 (PP: APP: CFA = 78 : 17.6 : 4.4) sample shows significant changes. The PHRR of PP/IFR decreased by 668.7 kW m^−2^, representing a 68.3% reduction, and the THR decreased by 40.7 MJ m^−2^, representing a 27.0% reduction. The above results indicate that the maximum heat release rate of the material during combustion is reduced, the intensity of flame spread is weakened, the severity of combustion is decreased, and the total heat released during the entire combustion process is reduced, thereby lowering the fire hazard. Despite the shortened TTI by 9 s and *t*_PHRR_ by 127 s, indicating that the material is more easily ignited when exposed to heat and the initial combustion reaction is accelerated, this may be related to the early decomposition of the intumescent flame retardant, releasing a small amount of combustible gas or ammonia, along with rapid char formation. Combined with the reduction in PHRR and THR, it still effectively demonstrates overall flame retardancy, further indicating that the primary role of the flame retardant is to inhibit the spread of combustion rather than delay ignition. The increase in FPI and a decrease in FGI for PP/IFR indicate an improvement in the material's flashover resistance and a reduction in fire hazard; meanwhile, a reduction in TSR by 996.7 m^2^ m^−2^ suggests enhanced smoke suppression capability of the material. Further increasing the IFR content (PP: APP: CFA = 78 : 20.8 : 5.2) can better improve the heat release performance and reduce fire hazard, but the smoke suppression ability is slightly weaker. In summary, when PP/IFR is heated, the APP in IFR releases phosphorus-containing inorganic acids as dehydrating agents, promoting the cross-linking of the matrix into char. The thermal decomposition of CFA releases non-combustible gases, causing the molten material in the early stage of carbonization to expand and form a dense, porous, foam-like char layer (Fig. 4). This layer acts as a thermal insulation, oxygen barrier, and barrier to combustible gases, preventing PP combustion by depriving it of sufficient fuel and oxygen supply, thereby achieving flame retardancy and also inhibiting smoke generation.

**Table 5:** Cone calorimeter test data of PP and its flame-retardant composites

Sample	TTI [s]	PHRR [kW m^−2^]	*t*_PHRR_ [s]	THR [MJ m^−2^]	FPI [s m^2^ kW^−1^]	FGI [kW (m^−2^ s^−1^)]	TSR [m^2^ m^−2^]
PP	43	978.5	200	150.5	0.044	4.89	1451.4
P1	34	309.8	73	109.8	0.109	4.24	454.7
P2	27	255.8	71	113.8	0.106	3.60	769.6
P6	29	251.1	65	121.9	0.115	3.86	233.3

Note: TTI refers to the Time to Ignition, which is the time required for a sample to be continuously ignited by a standard electrical arc ignition source after being exposed to a radiant heat source with a heat flux of 35 Kw m^−2^; PHRR is the maximum heat release rate of the sample during combustion; *t*_PHRR_ represents the time to peak heat release rate, which is the time required for the sample to reach the maximum heat release rate during combustion; THR is the Total Heat Release, which is the total amount of heat released by the sample throughout the entire combustion phase; FPI denotes the Fire Performance Index, which is the ratio of the ignition time to the peak heat release rate; FGI represents the Fire Growth Index, which is the ratio of the peak heat release rate to the time to peak heat release rate; TSR is the cumulative amount of smoke produced by the sample during the entire combustion phase.

By introducing varying amounts of homemade synergistic flame retardant BAAEP into the PP/IFR (PP: APP: CFA = 78 : 17.6 : 4.4) system, the above situation can be significantly improved. When the BAAEP addition reaches 4 g (P6), compared to PP/IFR, TTI is shortened by 5 s, and PHRR is reduced by 58.7 kW m^−2^, representing a decrease of 18.9%. *t*_PHRR_ is shortened by 8 s, and THR increases by 12.1 MJ m^−2^, representing an increase of 11.0%. FPI increases, FGI decreases, and TSR decreases by 221.4 m^2^ m^−2^. Comprehensive analysis shows that the introduction of BAAEP significantly improves the flame retardancy of PP/IFR (significantly reducing PHRR and TSR, increasing FPI, and decreasing FGI). Despite the slight increase in THR and the shortening of TTI and *t*_PHRR_, this may be because BAAEP, due to its easily decomposable nature, may participate in combustion and release heat, leading to a slight increase in THR; however, it accelerates the early formation of a protective carbon layer, allowing the material to enter a stable combustion stage more quickly rather than undergoing intense combustion, resulting in the shortening of TTI and *t*_PHRR_. Comparing the char residue images (Fig. 4), it can also be observed that the addition of BAAEP promotes rapid char formation and synergizes with IFR to form a denser, more stable, and more expanded char layer, which isolates heat and combustible gases, delaying heat release. In summary, the synergist BAAEP exhibits a biphasic synergistic flame retardant mechanism: (1) in the condensed phase, it synergizes with IFR to form a denser and earlier expanded carbon layer, inhibiting heat and mass transfer. (2) In the gas phase, it interferes with the combustion chain reaction and releases inert gases into the gas phase. Throughout the entire combustion process, the two effects coordinate with each other, further reducing the heat release and smoke generation of the material during combustion and improving its flame retardancy and smoke suppression performance. The specific flame retardant mechanism still requires further verification.

#### Flame retardant mechanism

The flame retardant mechanism of the free radical scavenger was analyzed by examining the inner and outer char residues after cone calorimeter testing through SEM-EDX. As observed in Fig. S2, the outer char layer exhibits more wrinkles compared to the inner char layer. This is because, during the combustion process, the material in contact with air undergoes continuous thermal decomposition and gas release, leading to the gradual depletion of the substrate. This depletion creates a height difference with the cross-linked charred portion, resulting in the appearance of wrinkles. In addition, PP/IFR/BAAEP forms a denser char layer than PP/IFR, which is consistent with the results in Fig. 4.

**Fig. 6:**
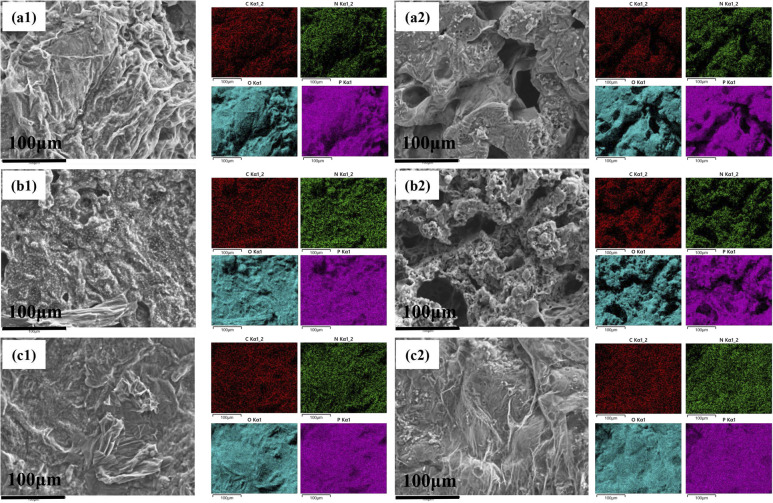
SEM images and EDX spectra of char residues after cone calorimeter tests: (a) P1; (b) P2; (c) P6; numbers 1–2 represent the outer char layer and inner char layer respectively.

**Fig. 7:**
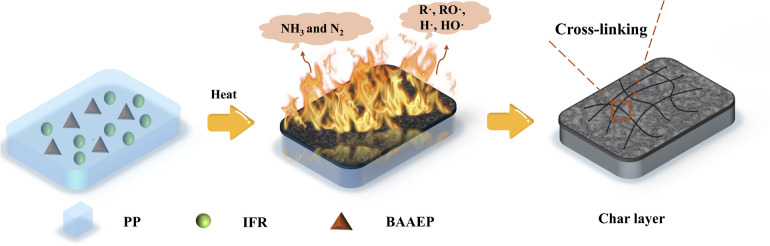
Flame retardant mechanism schematic illustration of PP/IFR BAAEP composites.

The variation in the element content of the char layers, both internal and external, provides compelling evidence for elucidating the flame-retardant mechanism (Fig. 6 and Table 6). It can be observed that the P content in the outer char layer is higher than that in the inner char layer for all three samples, while the N content in the outer char layer is lower, which reflects the intrinsic intumescent flame-retardant function, consistent with the results shown in Fig. 4. On the one hand, when the P element in the flame retardant primarily acts on PP through condensed-phase flame retardancy, the smaller the difference in P element content between the inner and outer char layers, as exemplified by the BAAEP/IFR/PP (P3) sample. On the other hand, as shown in Fig. 7, because the nitrogen-containing flame retardants such as APP, CFA or BAAEP synergists promote cross-linking and charring during pyrolysis, and may exist in the char layer as a nitrogen-containing heterocyclic structure to enhance thermal stability, resulting a higher *N* content in the inner char layer. In the formation of the outer char layer, CFA releases NH_3_ and N_2_, diluting oxygen and combustible gases through decomposition, while BAAEP captures active radicals (R·, RO·, H·, HO·) from PP decomposition in the gas phase by forming nitrogen–oxygen radicals, leading to the quenching of these active radicals and achieving a flame-retardant effect.

**Table 6:** The content of each carbon layer of residual carbon layer after conical calorimetry test of PP and its flame-retardant composite materials

Sample	Weight percentage (%)			
	P	C	O	N
P1 (outer)	23.93	17.24	55.32	3.51
P1 (inter)	13.71	10.98	65.61	4.84
P2 (outer)	24.83	11.35	59.30	4.52
P2 (inter)	18.73	20.91	53.53	6.83
P6 (outer)	24.04	11.96	60.33	3.67
P6 (inter)	22.02	12.17	60.84	4.97

Due to the incomplete combustion of PP in the presence of oxygen, the PP is not fully converted into CO_2_ and H_2_O, but instead participated in carbon chain rearrangement and branching within the matrix, forming C–O and C=O bonds, resulting in a significant amount of oxygen element in the residual carbon. In the PP/IFR (P1), the outer and inner char layers exhibit a pattern where the inner char layer has more O elements and fewer C elements compared to the outer char layer. This is because the outer char layer is fully exposed to air and can burn more completely than the inner char layer, which contains more incompletely burned materials and oxygen-containing substances such as P=O. When the amount of IFR is increased (P2), the O and C content in the outer and inner char layers show opposite trends, possibly due to excessive oxidative degradation by phosphoric acid, which disrupts the integrity of the char layer. Compared to P1, when the IFR content is kept constant and the synergist BAAEP is introduced, the C and O content in the inner and outer char layers tend to balance, indicating that the amide groups in BAAEP promote uniform cross-linking and char formation in the condensed phase, ultimately leading to an increase in the amount of residual char.

**Fig. 8:**
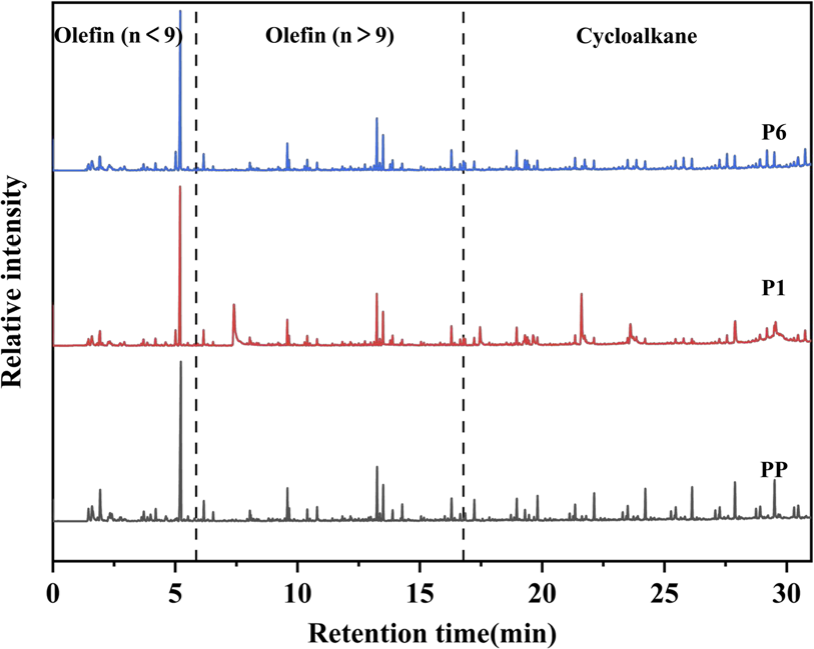
Py-GCMS spectrum of PP, P1, and P6.

The pyrolysis products of PP, PP/IFR, and PP/IFR/BAAEP, as well as the corresponding structures of the products, were analyzed by Py-GCMS (Fig. 8 and Tables S2–S4). It can be observed that after reaching the pyrolysis temperature, the main pyrolysis products of pure PP are olefins with carbon chains shorter than 9 (Olefin(*n* < 9)), olefins with carbon chains longer than 9 (Olefin(*n* > 9)), and cycloalkanes. The main products of PP/IFR and PP/IFR/BAAEP are also these three types of products. It is worth noting that the sulfur-containing compounds may be the sulfur elements introduced in the purchased APP and CFA. Through comparative analysis of Fig. 8, we found that PP/IFR and PP/IFR/BAAEP have significantly reduced cleavage products of olefins (*n* > 9) and cycloalkane compared to PP.

This may be due to the cross-linking of hydrocarbon chains with more than 9 carbon atoms with the matrix's carbon layer, making them more difficult to decompose. This also indicates that the char formation of the flame retardant can effectively protect the matrix at high temperatures and enhance the thermal stability of the matrix. The addition of BAAEP further reduces the content of cleavage products of olefins (*n* > 9) and cycloalkane, suggesting that the primary flame-retardant effect of BAAEP is on long-chain hydrocarbons and cycloalkane.

## Conclusions

This paper presents the preparation of a radical flame-retardant system (PP/IFR/BAAEEP) by the melt mixing method and the study of the synergistic flame-retardant effect of the radical scavenger BAAEEP with IFR. The thermal stability, mechanical and flame-retardant performance, and flame-retardant mechanism of the PP/IFR/BAAEEP system were analyzed.

This system can effectively enhance the thermal and thermo-oxidative stability of materials by catalyzing the cross-linking of the material matrix into carbon. Under nitrogen conditions, the *T*_max_ of PP/IFR/BAAEEP increased by 10 °C compared to pure PP, and the CY also improved significantly. In terms of mechanical performance, the introduction of the flame retardant IFR inevitably led to a decrease in the material's mechanical performance compared to pure PP. However, when the flame retardant synergist BAAEP was further added to improve the flame retardant performance, the component unexpectedly improved the interfacial compatibility between IFR and the matrix, and this synergistic effect compensated for the loss of mechanical performance caused by the addition of flame retardants.

The flame retardancy performance has improved significantly, with enhanced performance indices compared to pure PP. The PHRR of PP/IFR/BAAEP (with 4 g of BAAEP) was 251.1 kW m^−2^, which was a 74.34% decrease compared to PP. The *t*_PHRR_ has been reduced by 8 seconds, and the THR was 121.9 MJ m^−2^, which was a 19.0% decrease. The FPI has increased, while the FGI has decreased. The TSR was 233.3 m^2^ m^−2^, which was an 83.93% decrease. In addition to flame retardancy, smoke suppression was also achieved, upgrading the UL-94 combustion rating to V-0 and increasing the LOI to 32.8%. Research into flame-retardant mechanisms has found that, through multi-reactive groups such as amide, double bond, and amino groups, BAAEP catalyzes the cross-linking of the PP matrix into char while simultaneously scavenges gaseous free radicals generated from PP decomposition in the gas phase. Furthermore, combined with the intumescent charring effect of IFR, a physical and chemical synergistic flame-retardancy mechanism was established, achieving the integration of high-efficiency flame retardancy and smoke suppression. This study provides a new strategy for the development of high-performance PP with efficient flame retardancy, smoke suppression, and excellent mechanical performance.

## Author contributions

Xiao-han Zhou: conceptualization, data curation, formal analysis, investigation, methodology, writing – original draft, writing – review & editing. Xiao-ling Zang: data curation, formal analysis, writing – review & editing, visualization. Tian-chao Shi: data curation, visualization, writing – review & editing. Jing Xie: investigation. Ping-li Wang: investigation. Jun-hui Ji: supervision. Ge-xia Wang: project administration. Dan Huang: data curation, formal analysis, writing – original draft, writing – review & editing. Xu-ran Liu: funding acquisition, validation, writing – review & editing. Zhi-chao Zhen: conceptualization, methodology, writing – review & editing.

## Conflicts of interest

There are no conflicts to declare.

## Supplementary Material

RA-015-D5RA05200A-s001

## Data Availability

The data supporting this article have been included as part of the SI. Supplementary information: Fig. S1 – SEM images of PP and its flame-retardant composites; Fig. S2 – SEM images of char residue after cone calorimeter test. Table S1 – the elemental analysis data of flame retardant synergist BAAEP; Tables S2–S4 – the structure of main pyrolysis products of PP, P1 and P6 respectively. See DOI: https://doi.org/10.1039/d5ra05200a.

## References

[cit1] Hua L., Yun Y., Ying G., Juan D. (2022). Zhongwai Nengyuan.

[cit2] Bai G., Guo C., Li L. (2014). Constr. Build. Mater..

[cit3] Qiu L., Gao Y., Zhang C., Yan Q., O'Hare D., Wang Q. (2018). Dalton Trans..

[cit4] Luo Y., Xie D., Chen Y., Han T., Chen R., Sheng X., Mei Y. (2019). Polym. Degrad. Stab..

[cit5] Wang J., Ren Q., Zheng W., Zhai W. (2014). Ind. Eng. Chem. Res..

[cit6] Yan Y. W., Chen L., Jian R. K., Kong S., Wang Y. Z. (2012). Polym. Degrad. Stab..

[cit7] Schartel B., Wilkie C. A., Camino G. (2016). J. Fire Sci..

[cit8] Tu Z., Ou H., Ran Y., Xue H., Zhu F. (2024). J. Loss Prev. Process Ind..

[cit9] Zhou R., Lai X., Li H., Tang S., Zeng X. (2013). Polym. Compos..

[cit10] Tai Q., Shan X., Song L., Lo S., Yuen R. K. K., Hu Y. (2013). Polym. Compos..

[cit11] Lecouvet B., Sclavons M., Bailly C., Bourbigot S. (2013). Polym. Degrad. Stab..

[cit12] Huang J., Liang M., Feng C., Liu H. (2016). Polym. Eng. Sci..

[cit13] Li N., Xia Y., Mao Z. W., Wang L., Guan Y., Zheng A. (2013). Polym. Polym. Compos..

[cit14] Qian Y., Wei P., Jiang P., Hao J., Du J. (2013). Composites, Part B.

[cit15] Pappalardo S., Russo P., Acierno D., Rabe S., Schartel B. (2016). Eur. Polym. J..

[cit16] Tang Q., Yang R., Song Y., He J. (2014). Ind. Eng. Chem. Res..

[cit17] Kiliaris P., Papaspyrides C. D. (2010). Prog. Polym. Sci..

[cit18] Sai T., Ran S. Y., Guo Z. H., Song P. A., Fang Z. P. (2022). Sus. Mat..

[cit19] Katiyar R., Bag D. S., Nigam I. (2013). Thermochim. Acta.

[cit20] Pan Y., Guo Z., Ran S., Fang Z. (2019). J. Appl. Polym. Sci..

[cit21] Yuan B., Hu Y., Chen X., Shi Y., Niu Y., Zhang Y., He S., Dai H. (2017). Composites, Part A.

[cit22] Yuan B., Fan A., Yang M., Chen X., Hu Y., Bao C., Jiang S., Niu Y., Zhang Y., He S., Dai H. (2017). Polym. Degrad. Stab..

[cit23] Yang Y., Diaz Palencia J. L., Wang N., Jiang Y., Wang D. Y. (2021). Molecules.

[cit24] Yeoh G. H., De Cachinho Cordeiro I. M., Wang W., Wang C., Yuen A. C. Y., Chen T. B. Y., Vargas J. B., Mao G., Garbe U., Chua H. T. (2024). Adv. Mater..

[cit25] Xu S., Zhang M., Li S. Y., Zeng H. Y., Du J. Z., Chen C. R., Wu K., Tian X. Y., Pan Y. (2020). Appl. Clay Sci..

[cit26] Jia H., Onishi H., Wagner R., Winter M., Cekic-Laskovic I. (2018). ACS Appl. Mater. Interfaces.

[cit27] Pérez N., Qi X. L., Nie S., Acuña P., Chen M. J., Wang D. Y. (2019). Materials.

[cit28] Ma C., Li J. (2019). Composites, Part B.

[cit29] Kashani N. S. S., Gharavani F., Jaberi N., Tayouri M. I., Maleki F., Khonakdar H. A., Otadi M. (2021). Plast., Rubber Compos..

[cit30] Cao K., Wu S. l., Qiu S. l., Li Y., Yao Z. (2012). Ind. Eng. Chem. Res..

[cit31] Xie H., Lai X., Zhou R., Li H., Zhang Y., Zeng X., Guo J. (2015). Polym. Degrad. Stab..

[cit32] Lai X., Qiu J., Li H., Zhou R., Xie H., Zeng X. (2016). J. Anal. Appl. Pyrolysis.

[cit33] Nakashima E., Hosokawa Y., Ueno T. (2023). J. Appl. Polym. Sci..

[cit34] Ma C., Qian L., Li J. (2021). Polym. Degrad. Stab..

