# Supplementary material for: Designing high-performance polypropylene via synergistic free radical scavenging-intumescent flame retardancy: excellent mechanical performance and flame retardancy
Source: RSC Adv. 2025 Sep 22;15(41):34585–94. doi: 10.1039/d5ra05200a (PMC12451520; doi:10.1039/d5ra05200a)
Supplement: RA-015-D5RA05200A-s001 [file RA-015-D5RA05200A-s001.pdf]

Supplementary Information (SI) for RSC Advances.
This journal is © The Royal Society of Chemistry 2025

Supplementary Information:

Table S1. The elemental analysis data of flame retardant synergist BAAEP

	Calculated value				Measured value			
Element	C	H	N	O	C	H	N	O
Content [%]	58.18	9.01	20.88	11.92	54.25	10.85	25.62	9.28

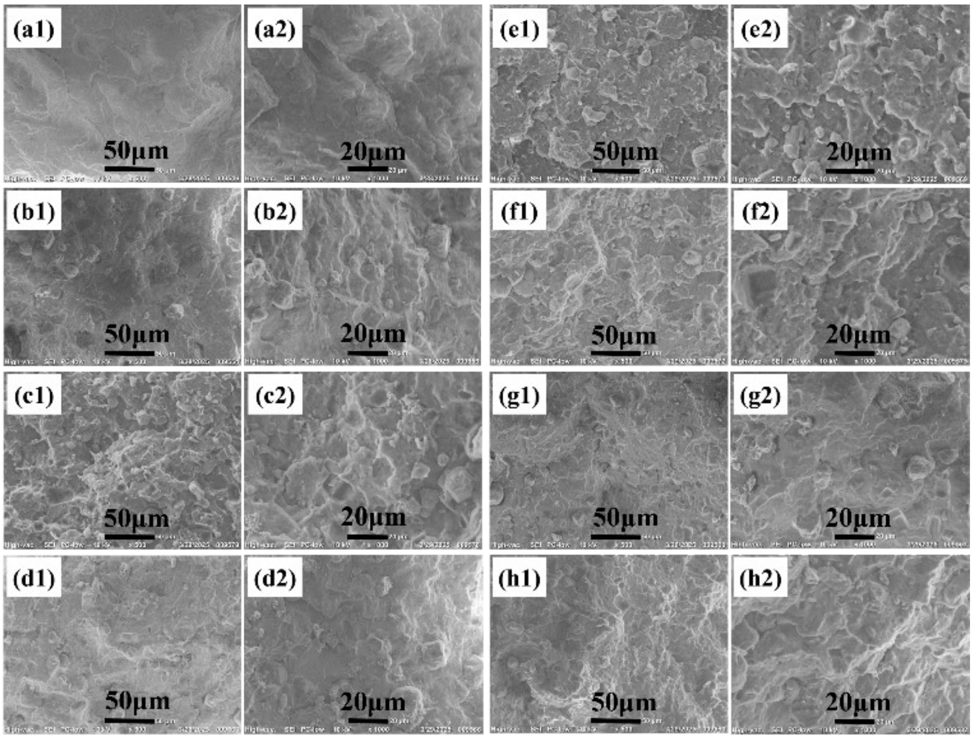

Fig. S1 SEM images of PP and its flame-retardant composites, where (a)-(h) represent samples PP, P1-P7 respectively; numbers 1 and 2 indicate magnifications of 500 and 1000, corresponding to scale bars of 50 μm and 20 μm, respectively.

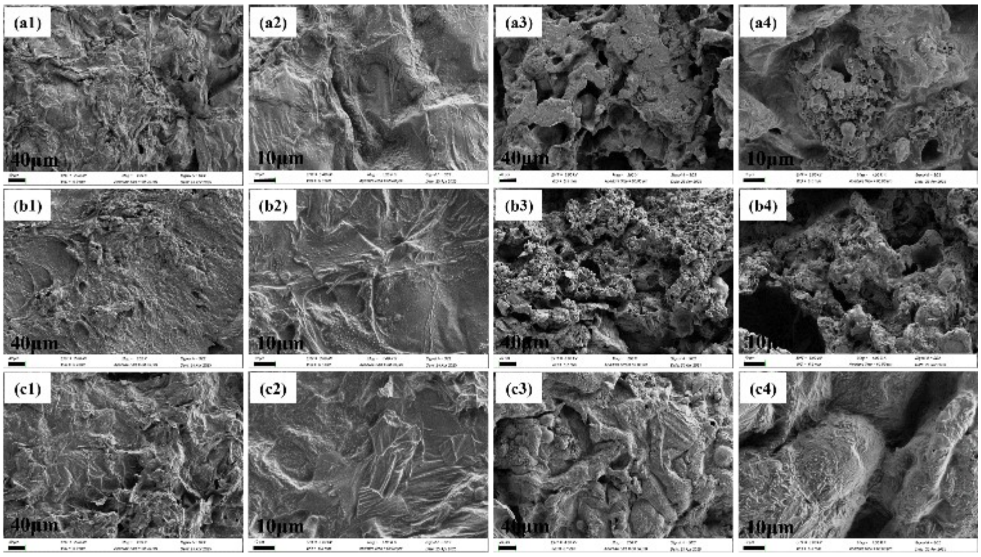

Fig. S2 SEM images of char residue after cone calorimeter test: (a) P1; (b) P2; (c) P6;

numbers 1-2 represent the outer char layer at 500x and 1000x magnification, respectively, and numbers 3-4 represent the inner char layer at 500x and 1000x magnification, respectively.

Table S2. The structure of the main pyrolysis products of PP was tested by Py-GCMS.

Peak area (%)	Retention time (min)	Assigned structure
1.61	1.45	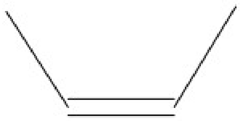
2.63	1.60	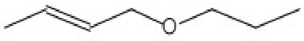
3.83	1.93	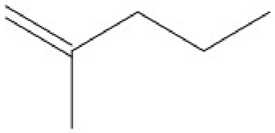
20.03	5.23	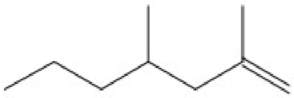
1.64	6.17	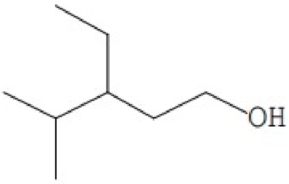
2.54	9.59	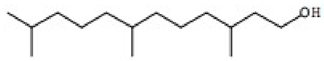
4.57	13.25	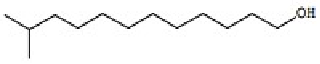
1.99	16.30	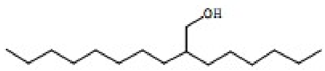
1.80	17.23	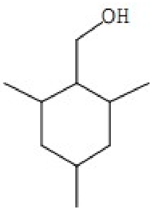

1.95	18.96	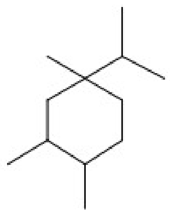
3.82	19.50	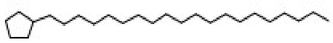
2.94	26.13	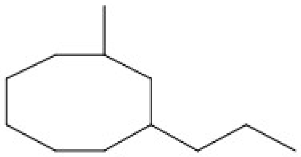
3.82	29.50	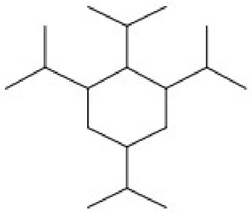

Table S3. The structure of the main pyrolysis products of P1 was tested by Py-GCMS.

Peak area (%)	Retention time (min)	Assigned structure
1.60	1.60	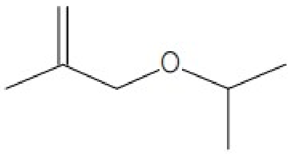
2.06	1.92	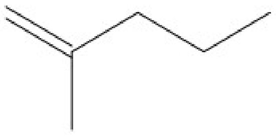
1.25	5.01	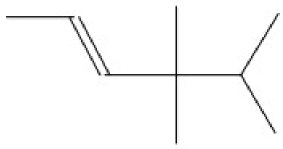
15.43	5.20	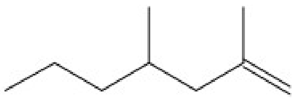

9.06	7.40	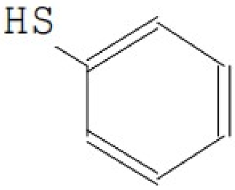
1.89	9.58	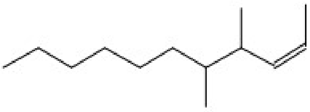
3.98	13.24	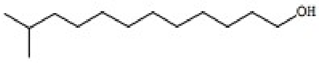
2.18	17.46	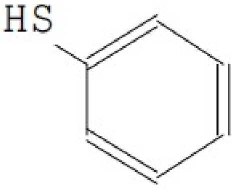
1.92	19.63	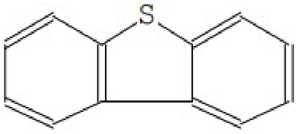
7.73	21.61	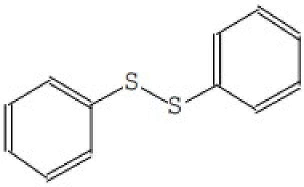
3.70	23.61	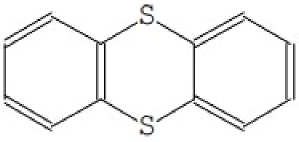
3.61	27.87	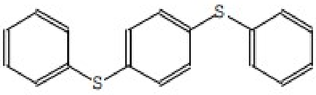
1.77	29.54	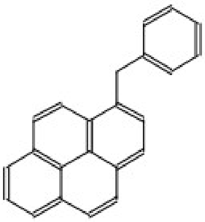

2.28	31.09	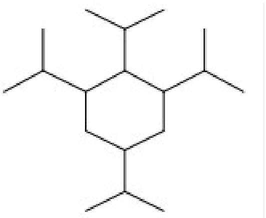

Table S4. The structure of the main pyrolysis products of P6 was tested by Py-GCMS.

Peak area (%)	Retention time (min)	Assigned structure
2.25	1.60	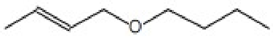
2.64	1.92	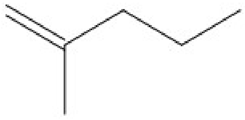
2.28	5.02	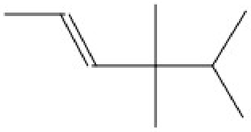
18.77	5.20	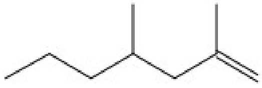
1.63	6.16	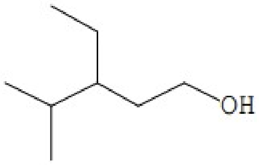
2.28	9.58	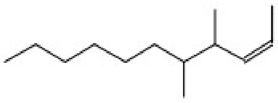
4.76	13.24	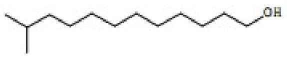
1.81	29.19	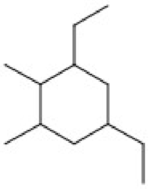
1.94	30.75	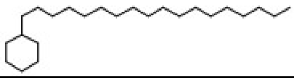
